# BOLD Temporal Dynamics of Rat Superior Colliculus and Lateral Geniculate Nucleus following Short Duration Visual Stimulation

**DOI:** 10.1371/journal.pone.0018914

**Published:** 2011-04-29

**Authors:** Condon Lau, Iris Y. Zhou, Matthew M. Cheung, Kevin C. Chan, Ed X. Wu

**Affiliations:** 1 Laboratory of Biomedical Imaging and Signal Processing, The University of Hong Kong, Pokfulam, Hong Kong, Special Administrative Region, China; 2 Department of Electrical and Electronic Engineering, The University of Hong Kong, Pokfulam, Hong Kong, Special Administrative Region, China; 3 Department of Anatomy, The University of Hong Kong, Pokfulam, Hong Kong, Special Administrative Region, China; 4 Department of Medicine, The University of Hong Kong, Pokfulam, Hong Kong, Special Administrative Region, China; National Institutes of Health, United States of America

## Abstract

**Background:**

The superior colliculus (SC) and lateral geniculate nucleus (LGN) are important subcortical structures for vision. Much of our understanding of vision was obtained using invasive and small field of view (FOV) techniques. In this study, we use non-invasive, large FOV blood oxygenation level-dependent (BOLD) fMRI to measure the SC and LGN's response temporal dynamics following short duration (1 s) visual stimulation.

**Methodology/Principal Findings:**

Experiments are performed at 7 tesla on Sprague Dawley rats stimulated in one eye with flashing light. Gradient-echo and spin-echo sequences are used to provide complementary information. An anatomical image is acquired from one rat after injection of monocrystalline iron oxide nanoparticles (MION), a blood vessel contrast agent. BOLD responses are concentrated in the contralateral SC and LGN. The SC BOLD signal measured with gradient-echo rises to 50% of maximum amplitude (PEAK) 0.2±0.2 s before the LGN signal (p<0.05). The LGN signal returns to 50% of PEAK 1.4±1.2 s before the SC signal (p<0.05). These results indicate the SC signal rises faster than the LGN signal but settles slower. Spin-echo results support these findings. The post-MION image shows the SC and LGN lie beneath large blood vessels. This subcortical vasculature is similar to that in the cortex, which also lies beneath large vessels. The LGN lies closer to the large vessels than much of the SC.

**Conclusions/Significance:**

The differences in response timing between SC and LGN are very similar to those between deep and shallow cortical layers following electrical stimulation, which are related to depth-dependent blood vessel dilation rates. This combined with the similarities in vasculature between subcortex and cortex suggest the SC and LGN timing differences are also related to depth-dependent dilation rates. This study shows for the first time that BOLD responses in the rat SC and LGN following short duration visual stimulation are temporally different.

## Introduction

In the rodent visual system, light from the external environment is focused by the cornea and lens onto the retina. The retina is composed of photosensitive retinal ganglion cells that project axons carrying information about the light, such as its color and spatial pattern. The axons from both eyes come together to form the optic nerves, which transmit information from the retina to the brain. The majority of these nerve fibers, 90–95% in rats [1], cross the midline to the opposite side of the brain. From there, fibers are known to project to the ventral and dorsal lateral geniculate nuclei (vLGN and dLGN, respectively), lateral posterior nucleus, pretectum, and superior colliculus (SC) [2]. The majority of retinal axons project to the superficial layers of the superior colliculus. It is involved in numerous functions related to responding to visual stimuli, including orienting the body to the stimulus [3] and guiding spatial movement using visual information [4]. Another structure receiving up to 37% of retinal projections is the dLGN of the thalamus [5]. It primarily serves as a relay station between the retina and the visual cortex (VC), where higher level processing takes place. Relay neurons in the dLGN receive signals from fast conducting Y-like retinal axons and in turn, send signals to the VC along their own fast conducting axons [2]. Together, the SC and LGN receive almost all direct projections from the rat retina.

Most of our understanding of the visual system has come from animal studies conducted with invasive, small field of view, and/or terminal measurement techniques such as electrical recordings, c-fos immunohistochemistry, and 2-deoxyglucose labeling. In comparison, blood oxygenation level-dependent (BOLD) functional magnetic resonance imaging (fMRI) is a non-invasive technique that can simultaneously examine a large field of view (FOV) with high spatial resolution [6]. In addition to fMRI, various other forms of nuclear magnetic resonance have been used to study vision, including diffusion imaging [7], manganese-enhanced MRI [8], [9], [10], magnetic resonance spectroscopy [11], and structural MRI [12]. BOLD imaging relies on the different magnetic properties of oxyhemoglobin and deoxyhemoglobin to indirectly measure neuronal activity [6]. Mostly conducted on human subjects, BOLD has also been applied to study visual functions in animal models, such as cats [13], primates [14], and rodents [15], [16]. These studies recorded regions of the brain activated by visual stimuli. However, to date, relatively few fMRI studies have been conducted on human subcortical structures, such as the SC, because of technical challenges stemming from its small size and deep position near the brainstem [17], [18], [19]. The rat SC occupies a significantly larger portion of the brain, is located closer to the skull, and receives a greater fraction of retinal projections. Thus, the rat is a more suitable mammalian model for functional imaging studies of the SC, and possibly the LGN, responding to visual stimuli.

Recent BOLD experiments in the rat somatosensory cortex have observed intrinsic response timing differences following forepaw stimulation [20], [21]. The BOLD response was observed to rise fastest in cortical layer 4 and slowest at the surface. However, less is known about BOLD spatiotemporal dynamics in the subcortex, which is beyond the reach of conventional optical imaging techniques in rats. In this study, we apply BOLD fMRI on Sprague Dawley (SD) rats to measure differences in response temporal dynamics between the SC and LGN following short duration (1 s) monocular visual stimulation. Experiments are conducted with both gradient-echo (GE) and spin-echo (SE) sequences to provide additional confirmation. Rodent visual fMRI experiments have traditionally been conducted with long duration block-design stimuli, which are optimal for identifying responsive brain regions [22]. In contrast, shorter duration stimuli are better for estimating the shape of the hemodynamic response and associated timing parameters. Observed differences may expand our understanding of the spatiotemporal heterogeneities in BOLD responses in subcortical regions. This study represents the first measurements of BOLD response temporal dynamics in the rodent visual system using short duration stimulation.

## Results

**Figure 1:**
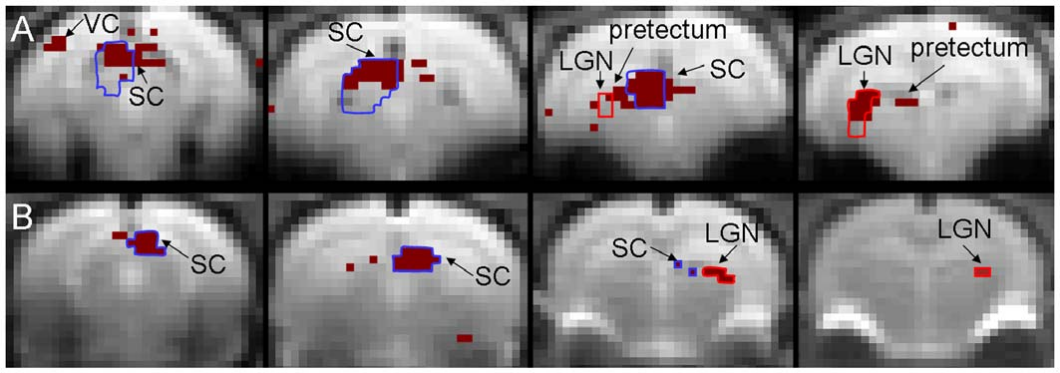
Gradient-echo and spin-echo activation maps. (A) Activation map computed by applying Analysis of Variance [23] on the four slice fMRI data (slices 1 to 4 arranged from left to right) of a representative animal scanned with the gradient-echo (GE) sequence. Voxels with p<10^−3^ are colored dark red. The blue and red regions of interest (ROIs) cover voxels containing the contralateral superior colliculus (SC) and lateral geniculate nucleus (LGN), respectively. The contralateral hemisphere is on the left. (B) Activation map computed from the average fMRI data of all animals scanned with the spin-echo (SE) sequence. Voxels with p = 0 (beyond computer precision) are colored dark red. Blue and red ROIs cover such voxels in the contralateral SC and LGN, respectively. The contralateral hemisphere is on the right. Low p-value regions of the SC, LGN, pretectum, and visual cortex (VC) are labeled.

Figure 1a shows the activation map measured from a representative animal using gradient-echo. Active voxels here are defined by p<0.001 as computed by Analysis of Variance [23]. Clusters of activation can be observed in the contralateral (left hemisphere) SC, VC, pretectum, and LGN. Some active voxels are also observed in the ipsilateral SC. Amongst the visual centers, the SC and LGN have the greatest density of active voxels. Figure 1b shows the activation map (p = 0) from the average data set of all spin-echo animals. Clusters of activation can again be observed in the contralateral (right hemisphere) SC, contralateral LGN, and ipsilateral SC. For both acquisition methods, SC active voxels are mostly in the superficial half, but some intermediate/deep voxels have p<0.001 when measured with GE.

**Figure 2:**
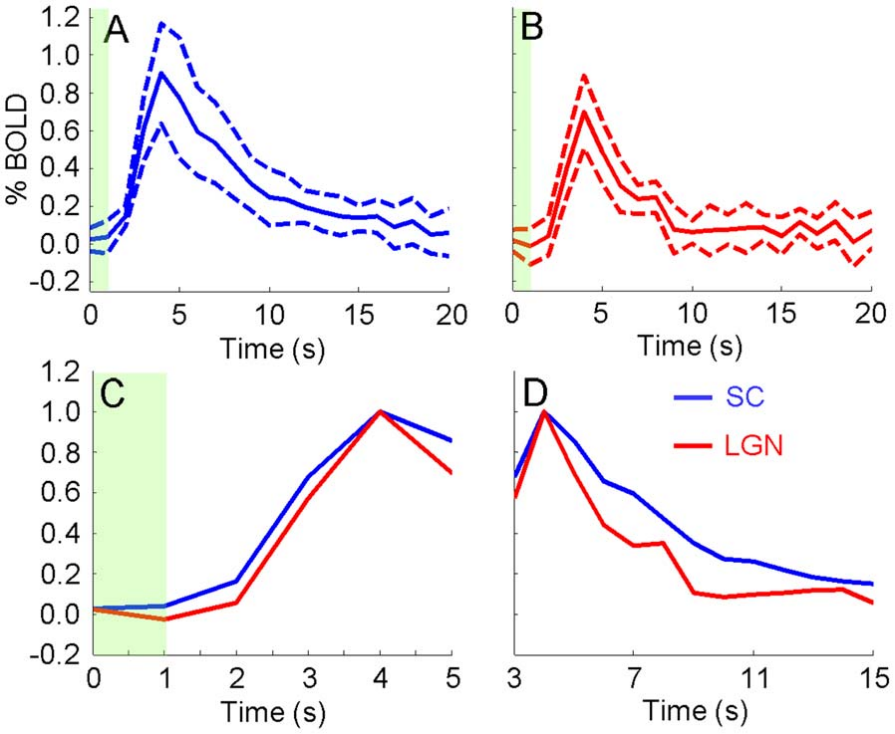
Gradient-echo BOLD signals. Mean (solid line) ± standard deviation (dashed line), computed across all animals scanned with GE, of BOLD signals from the SC (A) and LGN (B) ROIs in Fig. 1a. (C and D) Solid lines in (A) and (B) normalized to maximum amplitude of 1. The time axes indicate time from onset of stimulation. The green bars indicate the 1 s stimulation period.

**Figure 3:**
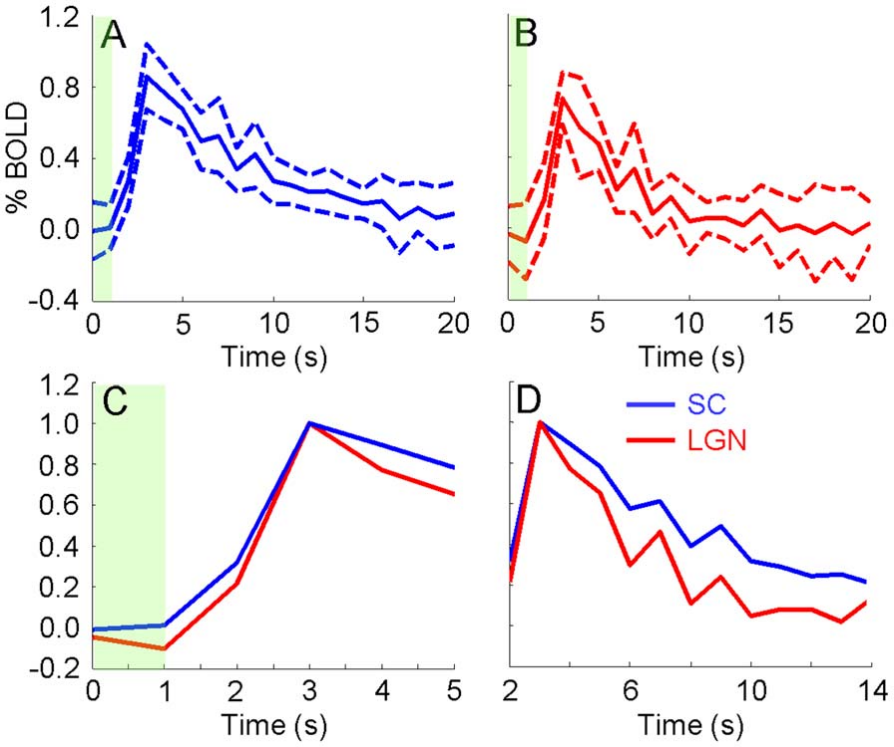
Spin-echo BOLD signals. Mean (solid line) ± standard deviation (dashed line), computed across all animals scanned with SE, of BOLD signals from the SC (A) and LGN (B) ROIs in Fig. 1b. (C and D) Solid lines in (A) and (B) normalized to maximum amplitude of 1. The time axes indicate time from onset of stimulation. The green bars indicate the 1 s stimulation period.

Figure 2 shows the mean and standard deviation of BOLD signals (computed across all animals scanned with GE) from all voxels in the SC and LGN regions of interest (ROIs) in Fig. 1a. In Fig. 2a and 2b, the responses in both regions rise significantly 2–3 s after onset of stimulation and reach maximum amplitude (PEAK) at 4 s. Both responses gradually return to baseline after reaching PEAK with the LGN response appearing to return faster. Figure 2c illustrates the temporal differences in the rising portions of the SC and LGN responses using the normalized mean BOLD signals. The SC response rises earlier than the LGN response. A small “initial dip” may be present in the LGN response. Figure 2d illustrates temporal differences in the falling portions of the responses. The LGN response approaches baseline faster than the SC response. Figure 3 shows the mean and standard deviation of BOLD signals (computed across all animals scanned with SE) from the SC and LGN ROIs in Fig. 1b. In Fig. 3a and 3b, the responses in both regions rise significantly approximately 2 s after onset of stimulation and reach PEAK at 3 s. Figure 3c shows the SC response rises earlier than the LGN response. A small “initial dip” may be present in the LGN response. Figure 3d shows the LGN response approaches baseline faster than the SC response. BOLD signals measured with both gradient-echo and spin-echo show the SC signal rises faster than the LGN signal but is slower returning to baseline.

Table 1 shows the mean and standard deviation of t50 and t150 measured from the animals scanned with GE. The SC BOLD signal reaches 50% of PEAK 2.6±0.1 s after onset of stimulation while the LGN signal requires 2.9±0.2 s. The difference is 0.2±0.2 s and statistically significant (p<0.05). The time to return to 50% of PEAK is 7.5±1.1 s for the SC and 6.1±1.2 s for the LGN. The difference is 1.4±1.2 s (p<0.05). Table 2 shows the mean and standard deviation of the areas under the rising and falling portions of the normalized BOLD signal (rAUS and fAUS, respectively) measured from the animals scanned with GE. rAUS from the SC is 0.8±0.1 and that from the LGN is 0.6±0.3. The difference is 0.2±0.3 (p<0.05). Along the falling portion, fAUS is 4.5±1.5 for the SC and 2.4±0.6 for the LGN. The difference is 2.0±1.5 (p<0.05). Table 3 shows the mean and standard deviation of rAUS and fAUS measured from the animals scanned with SE. rAUS from the SC is 0.3±0.2 and that from the LGN is 0.2±0.2. The difference is 0.1±0.1 (p<0.05). Along the falling portion, fAUS is 6.0±1.7 for the SC and 2.7±1.5 for the LGN. The difference is 3.4±2.1 (p<0.01). Together, these parameters quantify the temporal differences between the SC and LGN responses observed in Figs. 2 and 3.

**Table 1:** t50 and t150 measured from SC and LGN.

GE	t50 (s)	t150 (s)
**SC**	2.6±0.1	7.5±1.1
**LGN**	2.9±0.2	6.1±1.2
**SC – LGN**	−0.2±0.2*	1.4±1.2*

Mean and standard deviation (across all animals scanned with GE) of time to rise to 50% of PEAK (t50) and time to return to 50% of PEAK (t150) computed from SC and LGN ROIs in Fig. 1a. The differences between measurements from the SC and LGN are also shown.

*indicates statistical significance at the p<0.05 level (paired, two-tailed t-test).

**Table 2:** rAUS and fAUS measured from SC and LGN using GE.

GE	rAUS	fAUS
**SC**	0.8±0.1	4.5±1.5
**LGN**	0.6±0.3	2.4±0.6
**SC – LGN**	0.2±0.3*	2.0±1.5*

Mean and standard deviation (across all animals scanned with GE) of areas under the rising (rAUS) and falling (fAUS) portions of the signal computed from SC and LGN ROIs in Fig. 1a. The differences between measurements from the SC and LGN are also shown.

*indicates statistical significance at the p<0.05 level.

**Table 3:** rAUS and fAUS measured from SC and LGN using SE.

SE	rAUS	fAUS
**SC**	0.3±0.2	6.0±1.7
**LGN**	0.2±0.2	2.7±1.5
**SC – LGN**	0.1±0.1*	3.4±2.1**

Mean and standard deviation (across all animals scanned with SE) of rAUS and fAUS computed from SC and LGN ROIs in Fig. 1b. The differences between measurements from the SC and LGN are also shown.

* and ** indicate statistical significance at p<0.05 and 0.01 levels, respectively.

**Figure 4:**
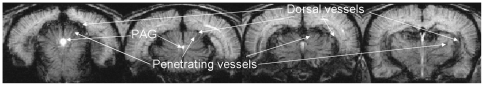
Post-MION FLASH images. Fast Low Angle SHot (FLASH) image (slices 1 to 4 arranged from left to right) acquired after intravenous injection of monocrystalline iron oxide nanoparticles (MION). The locations of large blood vessels dorsal of the subcortex and penetrating vessels are indicated. The periaquaductal gray (PAG) at the center of the midbrain is also indicated.

Figure 4 shows the Fast Low Angle SHot (FLASH) image acquired from one animal after injection of monocrystalline iron oxide nanoparticles (MION). The SC contains numerous thin dark lines running from the dorsal surface to the periaquaductal gray. These are likely penetrating blood vessels as iron oxide particles enlarge susceptibility differences between vessels and surrounding tissue [24]. Similar penetrating vessels also innervate the cortex. The responsive LGN regions appear to be closer to the large vessels dorsal of the subcortex than the majority of responsive SC regions due to the latter's larger size. Thin penetrating vessels are not as apparent in the LGN.

## Discussion

Significant responses are observed in the SC and LGN following visual stimulation. The SC BOLD signal is seen to rise faster and settle slower than the LGN signal. These findings are independent of the pulse sequence used. Using gradient-echo, the times to reach 50% of maximum BOLD response after onset of stimulation are 2.6±0.1 s for the SC and 2.9±0.2 s for the LGN. The times to return to 50% of maximum response are 7.5±1.1 s for the SC and 6.1±1.2 s for the LGN. rAUS and fAUS for the SC are 0.8±0.1 and 4.5±1.5, respectively. The values for the LGN are 0.6±0.3 and 2.4±0.6, respectively. Using spin-echo, rAUS and fAUS for the SC are 0.3±0.2 and 6.0±1.7, respectively. The values for the LGN are 0.2±0.2 and 2.7±1.5, respectively. Together, these parameters quantitatively show the SC BOLD signal rises faster than that of the LGN but settles at a slower rate. A post-MION FLASH image shows the entire LGN is relatively close to the large vessels above the subcortex while most of the SC is further away and supplied by thin penetrating vessels.

### Visual fMRI studies

Previous rodent visual fMRI studies used block-design stimulation paradigms to identify responsive regions [16], [25], [26]. Van Camp et al. studied the responses of rats to monocular and binocular stimulation. They observed activation in the VC, SC, and flocculus-paraflocculus of the cerebellum [26]. However, their study did not report on any LGN response. Pawela et al. observed responses in various parts of the rat brain (SC, VC, dLGN, and lateral posterior nucleus) to monocular and binocular stimulation. The cortical responses observed in their study appear to span more of the VC than those in this study. This difference may be related to the choice of isofluorane rather than medetomidine anesthesia. Also, the shimming volume in our study was chosen to cover the subcortex and optimize signals from subcortical areas such as the SC and LGN. In Pawela et al.'s results section, they noted “there appeared to be a slightly longer delay in the BOLD impulse response for the cortical regions compared to the subcortical structures”, but no temporal differences were reported between the two subcortical structures SC and dLGN [25]. This discrepancy with our results could be due to the longer stimulus duration and significantly longer repetition time (TR) used in their study. Also, the contrast to noise ratio of BOLD signals in their study may be lower than in the present study because we average the signals from multiple experiments. Note lateral posterior nucleus responses may be present in slice 3 of Fig. 1a, but this structure is in close proximity to the LGN and SC and thus, responses may be affected by partial volume spillover. The predominantly contralateral responses observed in this study are expected as the vast majority of rat retinal projections target the contralateral hemisphere [1].

In a human fMRI study, Wall et al. studied the responses of the SC, LGN, and primary visual cortex to binocular stimulation. They found the SC response was best fit with hemodynamic response functions that peaked at 4 or 5 s after the onset of stimulation while LGN and cortical responses were best fit with hemodynamic response functions (HRFs) peaking at 6 s [18]. This finding agrees with our results in that the SC HRF appears to rise earlier than the LGN HRF. However, it is not clear if their measured temporal differences can be quantitatively compared with the present results as the authors used significantly different processing steps due to a short inter-stimulus interval and the need to assume HRF shapes.

### Neuronal activity

The difference in temporal dynamics between BOLD signals from the SC and LGN observed in this study reflects differences in contributors to the BOLD signal. The increase in blood oxygenation responsible for BOLD contrast is due to a small increase in cerebral oxygen consumption accompanied by a larger increase in cerebral blood flow following neuronal activity [27]. Therefore, the BOLD temporal differences may be due to neuronal activity, neurovascular coupling, or hemodynamic response differences between the SC and LGN. Several studies had observed that neuronal activity in the SC affects activity in the LGN. Molotchnikoff et al. placed electrodes in the SC and LGN of rabbits and submitted them to flash visual stimulation [28]. First, the SC electrode measured spontaneous neuronal firing. A spike triggered the external flash after a set time delay and LGN firing was recorded. The delay was varied to study changes in LGN response with delay time. The authors found preceding SC activity reduced the amplitude of subsequent LGN activity from 0 to 250 ms after the initial spike. They also stated the latency of saccade is between 200–300 ms, suggesting the SC is involved with saccadic motion. Note the above study used spontaneous SC activation rather than stimulated activation. In a later set of studies, the same group first used a conditioning stimulus to fire SC neurons and followed after a set time delay with a test stimulus to fire LGN neurons [29]. They found the amplitude of LGN spikes changed after conditioning with the maximal change occurring 200–300 ms after SC firing. Changes were observed even when conditioning and test stimuli were presented simultaneously. Some of the spikes exhibited increased amplitude while a slightly larger number had decreased amplitude. This modification did not occur if the SC was inactivated by a localized injection of cobalt ions or potassium chloride. Similar findings have been observed in cats [30]. BOLD responses have been shown to correlate with the spiking activity measured above [31], although studies have shown BOLD is more closely correlated with low frequency local field potentials [32], [33]. In many experiments, spiking activity and local field potential vary in a similar manner [34]. Consequently, the BOLD temporal differences observed in this study may reflect the impact of SC activation on subsequent LGN activation. SC neuronal firing alters LGN firing in the proceeding 0 to 300 ms, leading to differences between the temporal dynamics and amplitudes of the BOLD signals. The 0.2±0.2 s t50 difference observed in this study is comparable to the time scales observed above.

### Vessel dilation rate

A second possible explanation of the findings of this study is based on differences in vessel dilation rate between the SC and LGN, independent of neuronal activation differences. During activation, the cerebral blood flow and cerebral oxygen consumption changes are followed by an increase in cerebral blood volume [27]. These hemodynamic changes help meet the energy demands of neuronal activity and have been related to the BOLD signal by analytical models [35], [36]. The models attribute the BOLD signal to total deoxyhemoglobin and blood volume changes resulting from expansion and relaxation of local blood vessels. Therefore, differences in vessel dilation rates between the SC and LGN can affect the temporal dynamics of their BOLD signals. Recently, Tian et al. used two-photon microscopy to observe differences in vessel dilation rates between the layers of the somatosensory cortex following electrical stimulation [21]. They measured the dilation rate of penetrating vessels that bring oxygen from surface vessels to capillary beds in the cortical layers and observed that the delay in vascular response decreased with depth down to the maximum penetration depth of the microscope. Branches off the main vessel trunks also dilated earlier at greater depths. The authors then compared the optical images to BOLD results and found that surface responses were delayed relative to responses from deeper layers, which agrees with an earlier study [20]. Combining measurements from the two modalities, the authors concluded differences in BOLD temporal dynamics with depth were related to differences in vessel dilation rates.

The vasculature pattern in the subcortex containing the SC and LGN is similar to that in the cortex. In the cortex, the large middle cerebral vessels lie on top (surface vessels above) while smaller penetrating vessels (arterioles above) supply the cortical layers [37]. In the subcortex, large vessels such as the supracollicular network lie dorsal of the SC and LGN while the different layers of the SC are supplied by smaller penetrating vessels (Fig. 4). Therefore, it is possible that neuronal activity in the subcortex will lead to a spatiotemporal pattern of vessel dilation similar to that in the cortex. The similarity between the BOLD signals in Figs. 2 and 3 of this study and signals from Tian's study support this extension. The LGN, which is closer to the dorsal vessels than most of the SC (Fig. 4), BOLD signal rises slower and settles faster. Similarly, BOLD signals from shallower cortical layers (Fig. 3 of Tian et al.) rise slower and also appear to settle faster compared to deeper layers [21]. We note that Tian et al. focused their study on the temporal differences in the rising portion of the signal and did not examine the return to baseline as closely. This similarity suggests the relative temporal dynamics between SC and LGN BOLD signals are related to depth dependent vessel dilation rate heterogeneities in the subcortex similar to those in the cortex.

The depth-dependent differences in vessel dilation rate may also explain why BOLD signals measured with gradient-echo reach PEAK later than those measured with spin-echo (Figs. 2 and 3). Compared to GE, SE is more sensitive to changes in small capillaries close to the site of neuronal activity and less to larger vessels such as those above the subcortex [38], [39]. As discussed earlier, dorsal vessels dilate after the smaller penetrating vessels and likely after the capillaries, meaning GE BOLD signals from a large ROI are expected to trail SE signals.

### Technical considerations

Mechanical ventilation is not performed in this study to permit the animals to regulate their own blood gas levels at the expense of an increased likelihood of fMRI baseline signal instability due to physiological changes. These potential instabilities are not likely to affect the findings as we closely monitor multiple vital signs to detect large physiological changes. We note that some physiological parameters, such as blood gas levels, are not directly sampled by our setup and may impact fMRI signals. The averaging of signals from multiple experiments on an animal helps reduce the effects of any non-repetitive instabilities. Also, any remaining signal instabilities are present in both SC and LGN BOLD signals.

There are two likely reasons for the apparent proximity of active SC voxels in Fig. 1b to large vessels in Fig. 4. First, the superficial SC layers (approximately top 0.5 mm in adult rats) receive the majority of direct projections from the retina, as stated in the introduction section. Therefore, the strongest BOLD responses will likely be detected in these layers, which lie adjacent to the large vessels. The data in Fig. 1b of this study show voxels with low p values are concentrated in the superficial SC. Similar observations can be made in Fig. 3b of [25] and Fig. 1a of [26]. Second, MION significantly reduces the T_2_^*^ of blood vessels and surrounding tissue, which leads to darkening in FLASH images within and near vessels [40]. Further, a voxel may contain both vessels and tissue. The FLASH signal from such a voxel will also be reduced after MION injection. Consequently, the darkened regions in Fig. 4 may be larger than the actual blood vessels. Together, these reasons likely explain the close proximity, and possible overlap, of active SC voxels in Fig. 1b and dark areas in Fig. 4.

### Conclusion

BOLD fMRI with gradient-echo and spin-echo echo-planar imaging sequences both observe that the SC BOLD response rises faster and settles slower than the LGN signal following short duration visual stimulation. Post-MION FLASH images show the rat cortex and subcortex appear to share similar vasculature patterns. The BOLD spatiotemporal heterogeneity observed in this study is likely related to depth dependent differences in blood vessel dilation rate similar to those in the somatosensory cortex following electrical stimulation. However, other factors such as SC neuronal activity influencing LGN activity may also play a role.

## Materials and Methods

### Animal preparation

All aspects of this study were approved by the Committee on the Use of Live Animals inTeaching and Research (CULATR) of the University of Hong Kong (CULATR Number: 2041-09). Sixteen SD rats weighing between 250 and 300 g were used in this study. Each animal was anesthetized with 4% isofluorane (mixed with room air) for 5 minutes in a plastic anesthetizing box (Harvard Apparatus, Holliston, MA). Controlled dosages were provided by an isofluorane vaporizer (ISOTEC 4, SurgiVet, Waukesha, WI). Anesthesia was maintained with 1% isofluorane throughout the course of setup and scanning. Once sedated, animals were placed in the prone position on an animal bed (Bruker BioSpin, Germany) with a head restraint and tooth bar to restrict motion. A receive-only quadrature surface coil (Bruker BioSpin, Germany) was placed over the dorsal side of the head such that the coil was centered between the two ears. The entire assembly was placed inside a 7T MRI scanner (PharmaScan, Bruker BioSpin, Germany) and warm water was circulated within the holder while rectal temperature was monitored (SA Instruments, Stony Brook, NY). Respiration rate was monitored with a pressure sensor (SA Instruments, Stony Brook, NY) attached to the abdominal area. Heart rate and saturation of peripheral oxygen were monitored with a pulse oximeter (SA Instruments, Stony Brook, NY) attached to one of the hind-paws. Vital sign measurements were monitored in real time but were not available for post-processing.

### MRI protocol

**Figure 5:**
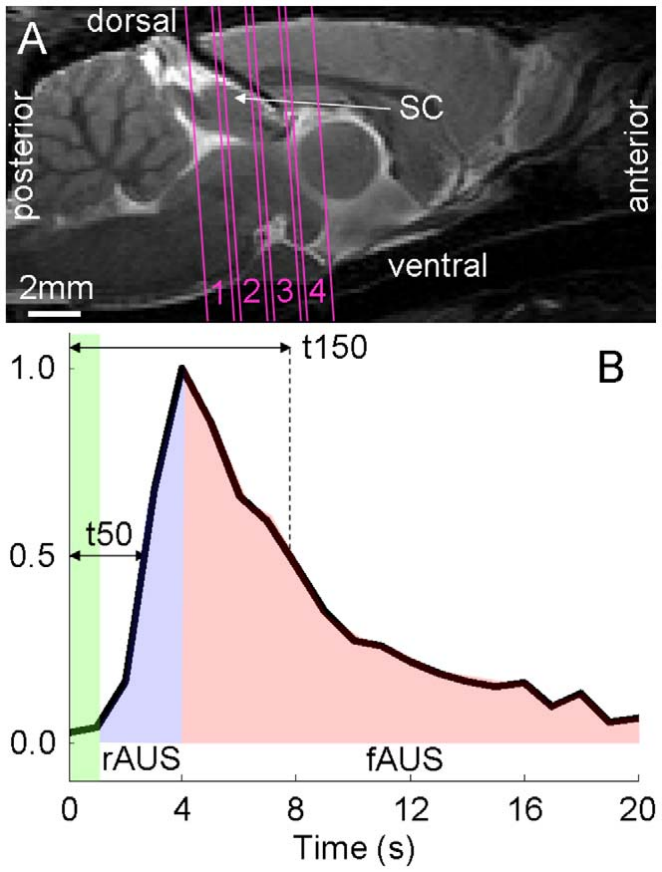
fMRI scan geometry and definition of parameters. (A) fMRI scan geometry overlaid on a sagittal scout image acquired at the midline of the brain. The four 1.0 mm thick scan slices, indicated by parallel solid lines and labeled 1 to 4, are oriented orthogonal to the sagittal plane as shown. The interslice gap is 0.2 mm. The location of the SC at midline is indicated. The anterior, posterior, dorsal, and ventral sides of the brain are also indicated. (B) Representative BOLD signal with maximum response amplitude (PEAK) normalized to 1. The times to rise to 50% of PEAK (t50) and to return to 50% of PEAK (t150) are defined. The areas under the rising (rAUS) and falling (fAUS) portions of the signal are defined by the areas shaded in blue and red, respectively. The green bar indicates the 1 s stimulation period.

Once the animal was properly positioned in the scanner, scout images were acquired to determine the coronal and sagittal planes. Four parallel 1.0 mm thick slices, separated by 0.2 mm, were oriented orthogonal to the sagittal plane as illustrated in Fig. 5(a) overlaid on an anatomical image of the brain at midline. According to the rat brain atlas of Paxinos and Watson [41], this geometry covered the SC and LGN and is similar to those employed in previous rodent fMRI studies [16], [25], [26].

An anatomical image was acquired from one animal with a Fast Low Angle SHot (FLASH) sequence (3.2 cm×3.2 cm, 256×256 voxels, TR = 250 ms, TE = 10 ms, α = 15°) after femoral vein injection of 15 mg Fe/Kg Monocrystalline Iron Oxide Nanoparticles (MION) [42], [43]. Iron oxide particles enlarge susceptibility differences between blood vessels and surrounding tissue, producing MRI contrast. For the BOLD experiments, the remaining animals were divided into two groups scanned with gradient-echo (GE, N = 7) and spin-echo (SE, N = 8) Echo-Planar Imaging (EPI) sequences. One animal from each group was chosen as a template for the group. Spin-echo sequences were used to complement the gradient-echo results as the SE signal is more selective to capillary responses close to the site of neuronal activity and less sensitive to the responses of large vessels [38], [39]. All animals were optically stimulated with a short stimulus duration paradigm adapted from rat somatosensory studies [44]. For gradient-echo, the paradigm consisted of five sets of 1 s stimulation followed by 60 s rest (inter-stimulus interval). An initial 10 s rest preceded the five sets. For spin-echo, the paradigm consisted of ten sets of 1 s stimulation with 25 s inter-stimulus interval. An initial 10 s rest also preceded the ten sets. Visual stimulation was provided by an optical fiber placed 1 cm from an eye. The opposite eye was covered with opaque tape. All GE experiments stimulated the right eye along with three of the eight SE experiments. The proximal end of the fiber was illuminated by a green LED flashed at 10 Hz with a duty cycle of 0.5. The LED was positioned outside of the MRI scanner. Throughout an experiment, GE (3.2 cm×3.2 cm, 64×64 voxels, TR = 1.0 s, TE = 18 ms, α = 56°) or SE (3.2 cm×3.2 cm, 64×64 voxels, TR = 1.0 s, TE = 43 ms) EPI scans were acquired. All LED flashes and EPI scans were synchronized by triggers sent from a custom LabVIEW setup (National Instruments, Austin, TX). The experiment was repeated 10 to 15 times for each animal with longer than two minutes rest in between. A total of 88 gradient-echo experiments were conducted on seven rats and 120 spin-echo experiments on eight rats. If vital signs changed significantly during the course of an experiment, the experiment was stopped and restarted after at least a two minute interval.

### Data analysis

The EPI images from each experiment were corrected for slice timing differences using SPM5 and registered to the mean image of the first experiment using the rigid-body transformation of AIR5.2.5 [45]. All experiments from an animal were averaged to form one data set. Images from each data set were normalized to the mean image of the corresponding template rat using the affine transformation of AIR. In the GE group, the data set from each animal was analyzed using Analysis of Variance [23] to compute the probability, expressed in p values, of a brain region responding to the stimulus. Regions of Interest (ROIs) were drawn around the contralateral (left) SC and LGN of the template animal using the rat brain atlas (refer to Fig. 1a) and the average time series from all enclosed voxels computed. Voxels wholly or partially enclosed by the ROI boundaries were included in the average. In the SE group, voxels from right eye stimulated rats were matched with the corresponding voxel from left eye stimulated rats by flipping images of the former about the midline of the brain. The data sets from all animals were averaged and analyzed using Analysis of Variance. ROIs were drawn around voxels with p = 0 (beyond computer precision) in the contralateral (right) SC and LGN of the average data set (refer to Fig. 1b). One SC and one LGN average time series was computed for each animal by averaging time series from voxels in the ROIs. For all animals, average time series were transformed into BOLD signals (units of % BOLD) by averaging the responses from 5 s before to 20 s after the start of each 1 s stimulus and dividing by the amplitude from 5 s before to onset of stimulation. A normalized BOLD signal was also computed from each BOLD signal by dividing the amplitude at each time point by the maximum response amplitude (PEAK).

The temporal properties of BOLD signals from the SC and LGN of GE rats were quantified using strictly empirical measures by determining the time to reach 50% of PEAK after onset of stimulation (t50) and the time to return to 50% of PEAK (t150). Linear interpolation to 0.1 s temporal resolution was applied for these calculations. Temporal properties were also quantified by integrating the normalized BOLD signals along the time domain. The area under the rising portion of the signal (rAUS) was computed by summing the time points of the signal from cessation of stimulation to the time of PEAK. If a time point of either the SC or LGN signals had a negative value, that time point was excluded from the summation for both structures. Larger values of rAUS indicated the signal rose faster. Similarly, the area under the falling portion of the signal (fAUS) was computed by summing the data points of the normalized signal from time of PEAK to 20 s after onset of stimulation (5 s before the next 1 s stimulus). Larger values of fAUS indicated the signal fell slower. The definitions of all four parameters were illustrated in Fig. 5b. For SE rats, only rAUS and fAUS were computed due to the lower contrast to noise ratio of SE data [39]. Statistically significant differences between SC and LGN parameters were determined using paired, two-tailed t-tests. GE and SE animals were treated as two separate groups when performing t-tests.
